# Social needs and healthcare utilization in NICU graduates

**DOI:** 10.1038/s41372-024-02105-z

**Published:** 2024-09-13

**Authors:** Cecile L. Yama, Rachel G. Greenberg, Erika Johnson, Deesha D. Mago-Shah

**Affiliations:** 1 UCLA National Clinician Scholars Program, Los Angeles, CA USA; 2 Los Angeles County Department of Health Services, Los Angeles, CA USA; 3 Duke Clinical Research Institute, Durham, NC USA; 4 Department of Pediatrics, Duke University School of Medicine, Durham, NC USA

**Keywords:** Health services, Risk factors, Health care economics, Public health, Paediatrics

## Abstract

**Objective:**

Unplanned healthcare utilization after neonatal intensive care unit (NICU) discharge challenges families and healthcare systems. The impact of social needs on post-NICU healthcare utilization is underexplored. Our objective was to identify social needs among NICU graduates and examine associations between social needs and post-NICU healthcare utilization.

**Study design:**

A prospective cohort design was used to screen for social needs and track healthcare utilization among 112 NICU graduates attending a NICU follow-up clinic (2021–2022). Associations between social needs and healthcare utilization were analyzed using non-parametric statistical tests.

**Results:**

Of 112 patients screened, 20 (18%) had some social need. Infants with social needs experienced statistically significant higher rates of hospitalizations, overall encounters, and missed appointments.

**Conclusion:**

Social needs are associated with increased unplanned healthcare utilization and missed appointments. Addressing these needs during NICU follow-up may improve preventative care attendance and reduce unplanned healthcare use, leading to better outcomes for vulnerable infants and cost-savings for healthcare systems.

## Introduction

Infants requiring neonatal intensive care unit (NICU) care are more likely to experience increased healthcare utilization after discharge, causing stress for families and high costs for healthcare systems [1]. Clinical risk factors for increased healthcare utilization after a NICU stay include gestational age (with increased risk among very preterm and late preterm infants), bronchopulmonary dysplasia, and medical complexity [2]. Infants with Medicaid insurance also have increased frequency of readmissions; however, the drivers of this relationship are not well understood [2]. There is conflicting evidence as to whether race is associated with readmission rates [3–5]. Yet social and racial disparities, including black race (considered here as a proxy of structural racism), lower income, lower education, or living in an area with increased exposure to violence are associated with preterm birth and initial NICU hospitalization [6–8]. Despite abundant research on the clinical risk factors associated with readmissions after NICU care, and social risk factors associated with initial NICU hospitalization, there remains limited study on the effects of social needs on healthcare utilization in infants that have graduated from the NICU.

Social needs, also referred to in some studies as social risks, are associated with increased pediatric readmissions, emergency care utilization and decreased well child visits; addressing these needs is associated with increased preventative care in infants and decreased high cost healthcare use for some conditions, including asthma [9–12]. Social needs are an individual or family-level measure of social drivers of health (previously known as the social determinants of health), and include food, housing, transportation and utility insecurity among other needs. Only 23% of level 2–4 NICUs nationwide screen for social needs during inpatient stays, and the frequency of screening for social needs in NICU graduates after discharge is unknown, making it difficult to understand the impact of social needs on post-discharge healthcare utilization at the national scale [13]. The only existing study that has explored the relationship between social needs and postnatal healthcare utilization in premature infants has demonstrated that food insecurity prenatally is associated with increased hospitalizations in the first 6 months of life [14]. To our knowledge, no published study has explored other social needs such as housing or transportation insecurity in a NICU graduate population.

Evaluating associations between social needs and healthcare utilization in this population is important for several reasons. First, it serves as a proxy for understanding the inequities that contribute to disease exacerbation and associated poor neurodevelopmental outcomes in an extremely vulnerable population [15]. Addressing these inequities, and the barriers that families face in overcoming them, can have longstanding impacts on health and development [16–18]. Second, addressing social needs may increase preventative care and decrease the financial, health, and emotional burdens of unplanned healthcare utilization on families and healthcare systems [11, 19]. Our objective was to identify the social needs of NICU graduates in a NICU follow-up clinic and to evaluate healthcare utilization rates in patients with social needs, including both measures of unplanned healthcare utilization and missed appointments. Our secondary aim was to determine association patterns between specific social needs and unplanned healthcare utilization as well as missed appointments.

## Methods

### Participants

All infants on their first visit to the Special Infant Care Clinic (SICC), a NICU follow-up clinic at Duke University, were eligible for the study. Infants were screened for social needs using a standardized screening tool designed by the North Carolina Department of Health and Human Services (DHHS) between Sept 2021 and June 2022. The screener was either administered to caregivers in a written-form version during the intake process or verbally by a social worker, with all versions subsequently reviewed verbally by our social worker. Written screens were offered in English and Spanish with phone-interpreter translation provided for caregivers who spoke other languages. Patients without a completed screener were excluded. Data for all patients whose caregivers were screened (*N* = 112) were entered into a REDCap database. Institutional review board approval was obtained before conducting this research, and need for informed consent was waived, as screeners represented a new routine in clinical practice, and chart review presented minimal risk of harm to subjects.

NICU graduates eligible for this study were those that met criteria for follow-up in SICC after NICU discharge and who were screened at their first visit. NICU graduates were followed-up in SICC and included in this study if they had at least one of the following characteristics: (a) born less than 32 weeks gestation; (b) birth weight less than 1500 g; (c) small for gestational age; (d) chronic respiratory disease (such as from chronic lung disease, pulmonary hypertension, history of extracorporeal membrane oxygenation, congenital diaphragmatic hernia); (e) neurologic injury/malformation (such as hypoxic ischemic encephalopathy, hydrocephalus, seizures, meningitis, periventricular leukomalacia); (f) neonatal abstinence syndrome; and/or (g) any other condition that placed the infant at risk for growth or developmental impairment, including genetic differences. Additionally, a subset of patients discharged from the NICU, considered the most medically complex infants, were eligible for enrollment in the Transitional Medical Home (TMH) Program within SICC, which provided caregivers with 24/7 pager access to a medical provider from the NICU follow-up team. Only this subset of patients was eligible to participate in the “phone encounters” measured in this study. TMH eligible infants included in this study were those with at least one of the following: (a) gestational age (GA) ≤ 26 weeks; (b) birth weight ≤ 1000 g; (c) discharge with technology dependency (gastrostomy tube, nasogastric tube, glucometer, oxygen, tracheostomy, ventilator, ostomy); (d) hydrocortisone or neonatal abstinence medication wean; (e) discharge taking two or more medications for a single medical condition. In this paper, “medically complex” is defined as the population enrolled in the TMH.

### Instrument

The North Carolina Department of Health and Human Services (DHHS) “Social Determinants of Health (SDOH) Screening Questions” screening tool was utilized for this study [20]. This screening tool was designed with key stakeholders across North Carolina with an interest in the social determinants of health (a systemic perspective on social needs, now known as the social drivers of health) who identified four priority domains: food insecurity, housing instability, lack of transportation, interpersonal violence [20]. Questions included in the SDOH Screening Questions were modified and adapted from existing screening tools including the Hunger Vital Sign and the Protocol for Responding to Assessing Patients’ Assets, Risks, and Experiences (PRAPARE) assessment tool; questions were written at an accessible reading level; and standardized across 18 clinical settings [20]. For the purpose of this study, questions on interpersonal violence were omitted due to concerns regarding patient safety when documenting interpersonal violence in caregiver-accessible notes. The resulting screening tool included six questions that focused on three domains of need, including: two questions on food-security needs, three questions on housing-security needs/energy insecurity needs, and one question on transportation-security needs (Appendix).

### Exposure variables

Our main exposure was presence of any social need, defined as a “yes” response to any question regarding a social need on our screening tool. A “yes” response to any question within a social need category was counted as a social need of that resource.

### Outcome variables

Our primary outcomes were unplanned encounters per month after NICU discharge (including emergency department visits, hospitalizations, phone encounters and total encounters) and percentage of missed appointments (“no-show rate”) for patients with or without social needs. Our secondary outcome variables were these same measures of unplanned and missed healthcare utilization for specific social needs. All encounter frequencies were calculated by counting number of encounter type from NICU discharge to the end of the study period, and dividing by the number of months of this time period. Percentage of missed appointments is calculated by the electronic health record (EHR) and was recorded for the patient at the time of data collection. Because of the potential for type 1 error due to multiple comparisons, findings for secondary outcomes should be interpreted as exploratory. If a patient had an ED encounter followed by a hospitalization, each encounter was counted separately. Of note, telephone encounters were only accessible to the subset of complex patients enrolled in our transition medical home with 24/7 pager access, meaning rates of utilization for this outcome reflects only this sub-population.

### Statistical analysis

First, we described the specific social needs NICU graduates and the characteristics of infants with and without social needs in our sample; we also conducted Fisher’s exact tests to determine whether there were significant differences in baseline characteristics for infants with and without social needs. Second, we compared the average number of the four types of unplanned encounters per month and rate of missed appointments among infants with and without social needs using Mann-Whitney U-test. Last, we compared the average number of four types of unplanned encounters per month and rate of missed appointments among infants with specific social needs, as well as select clinical and social characteristics that may be confounders in the relationship between social needs and healthcare utilization, using Fisher’s exact test for categorical variables and Mann-Whitney U-test for continuous variables. We used Stata version 18.0 (College Station, TX) for analysis, and *P*-values < 0.05 were considered statistically significant.

## Results

Patients included in this study consisted of 112 infants screened out of 237 new patient visits that occurred during the duration of the study. However, not all of the new patient visits were eligible as some presented on days when our social worker was not available or represented cardiac follow-up patients not eligible for our study though listed for similar visit type that could not be distinguished from SICC visits using our EHR. Screened infants were followed for an average of 4.6 months (SD 2.8 months) from NICU discharge for their monthly unplanned healthcare utilization and missed appointment rate. This range of follow-up time was due to differences in time of birth relative to the timing of the end of the study period. Table 1 summarizes the specific social needs present in our cohort and the characteristics of patients with and without any social needs. Overall in our cohort, 58% (*N* = 65) of infants were female, 53% (*N* = 59) of infants were born ≤ 32 weeks, 92% (*N* = 103) of parent respondents were English-speaking. Most infants had only Medicaid insurance (58%, *N* = 64) and 29% (*N* = 32) were enrolled in the TMH and thus defined as having medical complexity, including 17% (*N* = 19) with medical technology use. Of the 18% (*N* = 20) of infants with social needs, 50% (*N* = 10) had food-security needs, 45% (*N* = 9) had housing-security/energy insecurity needs, and 65% (*N* = 13) had transportation-security needs. Among these infants with social needs, 50% (*N* = 10) had one need, 40% (*N* = 8) had two needs, and 10% (*N* = 2) had three needs. Infants with social needs were more often <=32 weeks, female, with Spanish speaking caregivers, and mothers <=30 years old, though none of these differences were statistically significant. The only significant difference between infants with and without social needs was insurance status; with 90% (*N* = 18) of infants with social needs being insured by Medicaid alone.

**Table 1:** Demographics of infants who completed social needs screener by presence or absence of social need.

Infant Characteristics	Social Needs Present (*N* = 20, 18%)	Social Needs Absent (*N* = 92, 82%)	*p*
Social Need, *n (%)*			
Food-Security	10 (50%)		
Housing-Security/Energy	9 (45%)		
Insecurity			
Transportation-Security	13 (65%)		
1 social need	10 (50%)		
2 social needs	8 (40%)		
3 social needs	2 (10%)		
Gestational Age, *n (%)*			0.62
< = 32 weeks	12 (60%)	47 (51%)	
>32 weeks	8 (40%)	45 (49%)	
Sex, *n (%)*			0.32
Female	14 (70%)	51 (55%)	
Male	6 (30%)	41 (45%)	
Preferred Language, *n (%)*			0.31
English	17 (85%)	86 (93%)	
Spanish	3 (15%)	5 (5%)	
Other	0 (0%)	1 (1%)	
Maternal Age, *n (%)*			0.09
>30	7 (35%)	53 (58%)	
< =30	13 (65%)	39 (42%)	
Medical Complexity^a^*, n (%)*			
Medical Home Eligible	8 (40%)	24 (26%)	0.28
Technology Use	6 (30%)	13 (14%)	0.10
Insurance, *n (%)*			0.001
Any Private^b^	2 (10%)	45 (49%)	
Medicaid	18 (90%)	46 (51%)	

^a^“Medical complexity” is defined here as infants eligible for our NICU clinic’s Transitional Medical Home; criteria are defined in the methods.

^b^“Any Private” insurance is defined as any private insurance, including patients with both private and Medicaid insurance.

In a comparison between infants of families with and without social needs, infants with any social need had a significantly higher number of total unplanned healthcare encounters per month, frequency of hospitalizations, and missed appointments (“percent no show”) **(**Table 2**)**. Patients with a food-security need, housing-security need/energy insecurity need, and transportation-security need all demonstrated a higher frequency of missed appointments compared to patients without those needs. Having transportation-security needs was associated with more frequent hospitalizations and total unplanned encounters. Medically complex patients demonstrated a higher rate of hospitalizations and total encounters, but they did not have higher frequencies of missed appointments. Medicaid insurance was associated with increased frequency of missed appointments, but not with other measures of unplanned healthcare utilization. Gestational age was not associated with differences in unplanned healthcare utilization or missed appointment rates in our population. There was no significant difference between groups in the frequency of ED visits and phone encounters.

**Table 2:** Average number of healthcare encounters per month since NICU discharge by social need or clinical characteristic (Means).

	Encounter Type (count/month, SD)									
Social Needs	Phone^1^	*p*	ED	*p*	Hospitalizations	*p*	Total	*p*	No-Show (%)	*p*
Any Social Need		0.22		0.15		0.02		0.01		<0.001
Present	1.56 (2.37)		0.16 (.30)		0.14 (.26)		0.92 (1.65)		12.50 (10.90)	
Absent	0.99 (2.07)		0.11 (.36)		0.07 (.24)		0.45 (1.47)		3.34 (5.24)	
Food-Security Need		0.07		0.34		0.64		0.15		0.003
Present	3.06 (3.47)		0.16 (.31)		0.15 (.34)		1.23 (2.21)		12.10 (8.67)	
Absent	0.93 (1.92)		0.11 (.35)		0.08 (.24)		0.47 (1.42)		4.30 (6.99)	
Housing-Security										
/Utilities-Insecurity Need		0.10		0.53		0.15		0.22		0.007
Present	--^2^		0.24 (.44)		0.18 (.35)		1.19 (2.28)		13.2 (12.0)	
Absent	0.94 (1.86)		0.11 (.34)		0.08 (.24)		0.48 (1.43)		4.3 (6.5)	
Transportation-Security										
Need		0.22		0.33		0.005		0.002		<0.001
Present	1.56 (2.37)		0.14 (.28)		0.13 (.19)		1.24 (1.93)		13.85 (11.78)	
Absent	0.99 (2.07)		0.12 (.36)		0.08 (.25)		0.44 (1.43)		3.82 (5.82)	
Clinical and Social Characteristics										
Preferred Language		0.62		0.74		0.18		0.63		0.62
English	0.97 (1.89)		0.12 (.36)		0.09 (.26)		0.51 (1.43)		4.97 (7.62)	
Other	3.50 (4.95)		0.06 (.11)		0 (0)		0.83 (2.32)		5.44 (5.83)	
Insurance		0.18		0.95		0.48		0.80		<0.001
Medicaid	0.80 (1.67)		0.13 (.41)		0.08 (.20)		0.50 (1.37)		7.68 (8.52)	
Any Private	1.98 (2.95)		0.10 (.25)		0.09 (.30)		0.58 (1.71)		1.46 (3.38)	
Medical Complexity				0.33		0.03		<0.001		0.11
Yes	--		0.21(.56)		0.17 (.35)		1.51 (2.56)		6.06 (8.18)	
No	--		0.08(.21)		0.05 (.18)		0.15 (.32)		4.53 (7.20)	
Gestational Age		0.20		0.98		0.76		0.42		0.19
<= 32 weeks	1.36 (2.21)		0.13 (.42)		0.10 (.28)		0.69 (1.84)		5.33 (7.40)	
> 32 weeks	0.74 (1.99)		0.11 (.25)		0.07 (.20)		0.36 (1.02)		4.65 (7.59)	

^a^Phone encounters per month were only calculated for subset of patients with medical complexity, assigned to the Transitional Medical Home program (described in the methods) and provided with 24/7 phone pager access.

^b^Too few data points exist in this subcategory to calculate the true average.

## Discussion

To our knowledge, this is the first study that examines associations between social needs, unplanned healthcare utilization and missed appointments in NICU graduates. Using a clinic cohort population, we found that patients with social needs had increased frequency of hospitalizations, overall encounters, and a higher percentage of missed scheduled appointments. Patients with transportation-security needs in particular had higher rates of several measures of unplanned healthcare utilization. These findings collectively suggest that social needs correlate with heightened unplanned healthcare utilization and reduced adherence to scheduled appointments, including both primary care and specialist visits, among NICU graduates. NICU follow-up is an opportunity to screen for, identify and address these needs. Doing so may increase patients’ ability to attend scheduled care, which may prevent high-cost unplanned healthcare utilization in vulnerable infants.

Children of families with social needs have poor health outcomes compared to their counterparts [14, 21]. The types of healthcare utilization that patients engage in may serve as a mediator in this relationship and an opportunity to interrupt the mechanisms that lead to disparities in child health outcomes. Notably, in our NICU graduate population, those with social needs demonstrated both increased hospitalizations and missed more scheduled appointments. Scheduled care is critical to maintaining the health of infants with multi-system medical issues and increased risk for poor growth and development. Children often miss care due to families rightful prioritization of social needs; for example, parents may miss appointments to attend work, allowing them to provide basic necessities for their children. Children frequently miss care because families prioritize essential social needs. For instance, parents may miss appointments to go to work, which is necessary to secure necessities for their children. Additionally, a family’s ability to attend appointments can be directly affected by unaddressed social needs, such as lack of reliable transportation, or housing instability that forces them to relocate away from healthcare facilities [22, 23]. Our finding that NICU graduates with higher social needs ultimately have increased rates of hospitalization may be due to their inability to attend preventative care, thereby exacerbating chronic illness. NICU follow-up clinics that combine social follow-up with standard developmental and medical follow-up may play an important role in improving long-term outcomes for infants requiring NICU care [24].

Our study builds on previous work that examined social drivers of rehospitalization in infants who have spent time in the NICU. Karvonen et al. found that Black and Hispanic preterm infants were more likely to be readmitted after discharge, and Morris et al. found low income and insurance status to be predictors of rehospitalization [3]. While these studies are important in identifying system and individual-level risk factors that contribute to rehospitalization, they are limited as they do not represent clear opportunities for intervention at the healthcare system-level. We provide new evidence showing that specific social needs are associated with unplanned healthcare utilization and missed appointments. This information can inform both policy and clinic-based interventions aimed at addressing family social needs, potentially altering healthcare utilization patterns, reducing costs for health systems and alleviating stress for families.

Our study is limited by the size of our sample, absence of data on race (due to poor race data quality in the EHR), difference in the size of study groups, and further limited size of some subgroups (particularly non-English speakers). Our outcome measures of unplanned healthcare utilization are based on what is available in our EHR; and while the EHR includes information on some hospitals outside our network, it does not include encounters from healthcare systems that do not participate in our health information exchange. Our study is limited to infants who attended our NICU follow-up clinic and completed screening and excludes those who never presented to care at our clinic and therefore were not screened or those that were not screened for other reasons (i.e. social worker availability). Lower rates of social needs in our NICU graduate population may be due to most infants being seen by an inpatient social worker prior to discharge, and because medically complex patients receive a check-in call to within the first month after discharge as a part of our TMH program; both present opportunities to address social needs prior to our screener. Additionally, our low social need rate may represent a low screening rate despite existing needs or a lower prevalence of social needs in our tertiary care center.

Due to the small sample size, we are not able to adjust for potential cofounding variables. Medical complexity may represent one confounder. While medically complex patients demonstrated a higher rate of hospitalizations and total encounters, they did not have higher clinic no-show percentages. Insurance status, which can be used as a measure of poverty, is another possible confounder that was found to be related to some measures of healthcare utilization. While Medicaid patients did have increased no-show rates relative to privately insured patients, it was not to the same degree as those with social needs, and there was no relationship between insurance status and other forms of utilization. While poverty and medical complexity may explain some of the relationship between social needs and unplanned and missed healthcare utilization, the patterns are not the same, and social needs may have a separate effect that must be further investigated with larger patient samples. It is also important to note that in the NICU discharge population, very low birthweight infants and those with prolonged lengths of stay qualify for Medicaid as primary or secondary insurance regardless of family income status.

In summary, we found that social needs were associated with increased unplanned healthcare utilization and missed appointments in NICU graduates. Our findings emphasize the importance of screening for social needs in this population as an opportunity to alter health trajectories for high-risk infants. While screening for social needs is becoming more widespread in pediatric clinics, less than one quarter of NICUs nationwide screen for social needs and there is no available data on national social needs screening in NICU follow-up clinics [13]. Some hospital systems and insurers have acknowledged that addressing social needs can shape health and have taken the next step to provide transportation, access to housing, or Medically Tailored Food [25, 26]. To better understand and address issues of equity in NICU care, efforts must be made to include social needs screens in the EHR of NICU follow-up clinics, in neonatal network data collection and in claims databases; this will allow researchers to assess patterns of healthcare utilization and related clinical outcomes in larger samples of NICU follow-up patients with social needs. Next steps would be to examine the most effective, patient-centered means of addressing these needs (i.e. how to best refer patients to resources), and testing the effect of referral on healthcare utilization in this unique population.

## Supplementary information

Social Needs Screener

## Data Availability

Data will not be made publicly available as it contains sensitive information that may be used to identify patients. De-identified data may be made available upon request if approved by our IRB.

## Code availability

Code availability can be made available on written request from the authors.
