# Supplementary material for: Social needs and healthcare utilization in NICU graduates
Source: J Perinatol. 2024 Sep 13;44(12):1732–7. doi: 10.1038/s41372-024-02105-z (PMC11606918; doi:10.1038/s41372-024-02105-z)
Supplement: Supplementary file 1 — Social Needs Screener [file 41372_2024_2105_MOESM1_ESM.docx]

**Supplementary Information:**

Questions in Social Needs Screener

|  | **Yes** | **No** | **Prefer Not to Answer** |
| --- | --- | --- | --- |
| **Food** |  |  |  |
| 1. Within the past 12 months, did you worry that your food would run out before you got money to buy more? |  |  |  |
| 1. Within the past 12 months, did the food you bought just not last and you didn’t have money to get more? |  |  |  |
| **Housing/Utilities** |  |  |  |
| 1. Within the past 12 months, have you ever stayed: outside, in a car, in a tent, in an overnight shelter, or temporarily in someone else’s home (i.e. couch-surfing)? |  |  |  |
| 1. Are you worried about losing your housing? |  |  |  |
| 1. Within the past 12 months, have you been unable to get utilities (heat, electricity) when it was really needed? |  |  |  |
| **Transportation** |  |  |  |
| 1. Within the past 12 months, has a lack of transportation kept you from medical appointments or from doing things needed for daily living? |  |  |  |
